# Exposure to Sublethal Concentrations of Lead (Pb) Affects Ecologically Relevant Behaviors in House Sparrows (*Passer domesticus*)

**DOI:** 10.1007/s00244-024-01062-0

**Published:** 2024-04-10

**Authors:** Joseph F. Di Liberto, Simon C. Griffith, Cara J. Hall, Alexandra S. Mendelsohn, John P. Swaddle

**Affiliations:** 1 Department of Ecology and Evolutionary Biology, University of California, Los Angeles, Los Angeles, CA USA; 2 Department of Biology, William & Mary, Williamsburg, VA USA; 3 School of Natural Sciences, Macquarie University, Sydney, NSW Australia; 4 Institute for Integrative Conservation, William & Mary, Williamsburg, VA USA

## Abstract

Global contamination of environments with lead (Pb) poses threats to many ecosystems and populations. While exposure to Pb is toxic at high concentrations, recent literature has shown that lower concentrations can also cause sublethal, deleterious effects. However, there remains relatively little causal investigation of how exposure to lower concentrations of environmental Pb affects ecologically important behaviors. Behaviors often represent first-line responses of an organism and its internal physiological, molecular, and genetic responses to a changing environment. Hence, better understanding how behaviors are influenced by pollutants such as Pb generates crucial information on how species are coping with the effects of pollution more broadly. To better understand the effects of sublethal Pb on behavior, we chronically exposed adult wild-caught, captive house sparrows (*Passer domesticus*) to Pb-exposed drinking water and quantified a suite of behavioral outcomes: takeoff flight performance, activity in a novel environment, and in-hand struggling and breathing rate while being handled by an experimenter. Compared to controls (un-exposed drinking water), sparrows exposed to environmentally relevant concentrations of Pb exhibited decreases in takeoff flight performance and reduced movements in a novel environment following 9–10 weeks of exposure. We interpret this suite of results to be consistent with Pb influencing fundamental neuro-muscular abilities, making it more difficult for exposed birds to mount faster movements and activities. It is likely that suppression of takeoff flight and reduced movements would increase the predation risk of similar birds in the wild; hence, we also conclude that the effects we observed could influence fitness outcomes for individuals and populations altering ecological interactions within more naturalistic settings.

**Supplementary Information:**

The online version contains supplementary material available at 10.1007/s00244-024-01062-0.

Lead (Pb) is a widely distributed and toxic heavy metal pollutant that poses a threat to humans and wildlife alike (Assi et al. 2016). While Pb is known to occur naturally in small quantities within the Earth’s crust, anthropogenic extraction and use of the metal have greatly increased over the past two centuries (Marx et al. 2016). Globally, Pb is commonly used in the manufacture of many products such as batteries, water pipes, paints, firearm ammunition, cable sheaths, and, until recently, and gasoline (Cheng and Hu 2010; Assi et al. 2016; Williams et al*.* 2018). The industrial manufacturing processes to make these products, as well as improper waste control, coal burning, water runoff, increasing urbanization, and the extraction of the metal itself (Cheng and Hu 2010), has polluted an increasing number of environments across the world with Pb, exposing a growing number of organisms to this toxicant.

Lead exposure to contaminated water, soil, dust, air, or food (Levin et al. 2008; Cheng and Hu 2010) adversely affects a range of physiological and neurological processes in organisms (Neal and Guilarte 2010; Franson and Pain 2011; Assi et al. 2016). Continuous or heightened exposure to Pb causes the accumulation of this pollutant in organisms’ tissues where it exacerbates these toxic effects, potentially leading to mortality (Franson and Pain 2011). In many cases, however, organisms are not typically exposed to fatal Pb concentrations (Pain et al. 2019). Instead, they are exposed to sublethal concentrations of Pb which might also cause deleterious effects for organisms.

Exposure to sublethal concentrations of Pb can impair many bodily systems. For example, exposure to Pb has been associated with increased oxidative stress biomarkers in red blood cells (Cid et al. 2018), digestive tissue (Lee et al. 2019), and reproductive organs (Vallverdú-Coll et al. 2016). Exposure to Pb has also been associated with decreased immune function (Miller et al. 1998; Lee et al. 2019, Vallverdú-Coll et al. 2019), altered stress or hypothalamic–pituitary–adrenal (HPA)-mediated responses (Baos et al. 2006; White et al. 2022; Zheng et al. 2022), decreases in muscular development (Hossain et al. 2016; Pain et al. 2019), and impaired bone formation (Gangoso et al. 2009; Ishii et al. 2018) in a variety of vertebrates. Notably, exposure to Pb degrades neural tissue and decreases the function of synaptic processes among neurons (Nixdorf et al. 1997; Zou et al. 2020), both of which appear to influence downstream cognitive abilities (Monchanin et al. 2021; Burger and Gochfeld 2000; Levin et al. 2008). Given the plethora of biological pathways influenced by exposure to this pollutant, we predict that Pb exposure would influence behavioral endpoints in organisms. However, there have been few studies of how exposure to sublethal concentrations of Pb influences ecologically important behaviors.

The behavior of an organism can serve as a bio-indicator of the condition and functioning of multiple internal systems (Scott and Sloman 2004). Furthermore, a change in behavior is often the first noticeable response to environmental change (Peterson et al. 2017). Hence, alteration of behaviors can be indicative of the capacity of individuals to cope with rapid environmental change (Hellou 2011) including a response to pollutant exposure (Peterson et al. 2017; Pain et al. 2019). Multiple studies have reported associations between changes in behaviors and exposure to sublethal concentrations of Pb in birds (Gorissen et al. 2005; Grunst et al. 2018; McClelland et al. 2019), fish (Weber 1993), amphibians (Chen et al. 2006), and insects (Mogren and Trumble 2010). However, these are largely observational studies and there is a lack of experimental evidence showing that exposure to sublethal Pb itself causes changes in behavior.

To better understand the causal relationships between exposure to sublethal Pb concentrations and changes in behavior, we experimentally exposed wild-caught adult house sparrows (*Passer domesticus*) to environmentally relevant concentrations of Pb in their drinking water in aviaries for 9–10 weeks and quantified escape takeoff flight, movement in a novel environment, and in-hand struggling behaviors and breathing rates while being handled. These behaviors likely influence or could be indicative of aspects of avian survival and fitness in more naturalistic settings (Lima 1993; Kluen et al. 2014; McCowan et al. 2015). We chose the house sparrow as an avian model as it is a sedentary, non-migratory, and globally distributed songbird that often lives in human-dominated landscapes (Swaileh and Sansur 2006) and hence, is often exposed to anthropogenic pollutants including Pb (Swaileh and Sansur 2006). The house sparrow is invasive and abundant in the USA, where this study was conducted, and the removal of house sparrows from the wild is unlikely to have detrimental ecological consequences.

Based on previous research, we hypothesized that exposed birds would suffer physiological and neurological impairments (Cid et al. 2018; Ishii et al. 2018; Zou et al. 2020) that would manifest in reduced takeoff performance and reduced movements while exploring a novel environment. While similar explanations might predict a reduced breathing rate or decreased struggles while handled by an experimenter, we also recognized that Pb-exposed birds have been known to exhibit altered stress responses (Baos et al. 2006; Zheng et al. 2022) and so may conversely exhibit a higher breathing rate or increased struggles. Hence, we had opposite predictions of breathing rate and struggling behavior outcomes depending on the underlying explanation.

## Methods—Procedures Common to all Studies

### House Sparrow Acquisition and General Housing

**Fig. 1:**
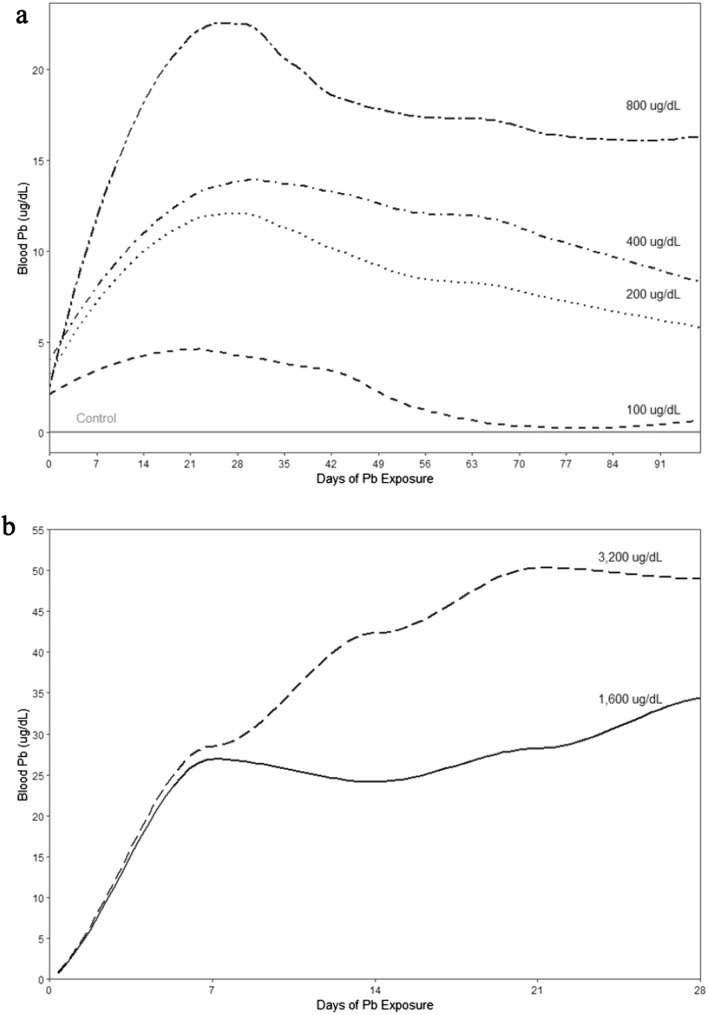
Average blood-Pb concentration over time of house sparrows exposed to Pb in **a** pilot study 1, and **b** pilot study 2. The different dashed/dotted lines represent a 2-point moving average over time so that the blood-Pb concentration of every bird in a treatment influenced this trajectory. Pb treatment concentrations are accordingly indicated above each line. The solid gray line in (**a**) represents the control treatment (No Pb in water). We elected to not add error bars because of the small sample size for each sample represented on the graph

We captured (with mist nets) adult house sparrows at five sites between Newport News, Williamsburg, and York counties in Virginia, USA (37.119382, − 76.465386) between February–March (pilot studies 1 and 2, *n* = 24) and September–November (main exposure study, *n* = 55) of 2022 (Fig. 1). Sites were chosen primarily based on the abundance of sparrows and their catchability. By choosing sites around residences or small businesses, we reasoned that the individual birds we caught would be likely to do well in captivity due to their familiarity with people and human-built structures. Once extracted from nets, birds were immediately transferred to a bird bag or holding bucket and transported to the nearby aviaries at William & Mary in Williamsburg.

Prior to treatment, birds were housed in outdoor aviaries (approximately 4L × 3W × 2.5H m) in groups of 12–18 birds with ad libitum access to food (50% Healthy Harvest chicken starter crumbs, 50% Volkman finch super seed blend) and oyster shell grit. We banded each bird with unique combinations of colored plastic hobby beads (Perler) on their tarsi so we could identify individuals. During December 2022, immediately before the start of the main study, we installed a heat lamp (Flukers’s Reptia-Clamp Lamp 8.5 in with 250W heat-emitting bulb) in each aviary to help the birds stay warm. These attributes of our aviaries have been known to maintain healthy populations of other avian species while standardizing the experience of captive birds undergoing similar behavioral assessments (Carlson et al. 2014). Since the birds were added to these groups relatively quickly across both our pilot and main study, we are doubtful that the shuffling of social groups would have strongly influenced the specific behaviors we studied. Daily human presence was consistent as a result of animal care, and due to this, it is possible that birds caught a few weeks earlier would be more acclimated to these disturbances. In general, we assume that all birds became somewhat habituated to human presence. These conditions and our study's procedures were held in accordance to William and Mary’s Institutional Animal Care and Use Committee (IACUC, protocol #2022-0017).

### Lead (Pb) Concentrations and Exposure in Drinking Water

To expose birds to Pb, we dissolved crystals of Pb acetate (Pb(C_2_H_3_O_2_)_2_) into their deionized (DI) drinking water. We placed two water dispensers in each aviary for the duration of each study. Each water dispenser contained 150 mL of either the control (no Pb), or Pb-exposed treatments. These dispensers were the only source of water available for the birds. Water dispensers had one small trough at the base so that birds could drink the water with minimal splashing/bathing in the dispenser. Once exposure began, we changed the treatment water every two days so the birds had continuous access to fresh control or Pb-exposed water for the duration of the experiment.

### Measuring Blood-Pb with the LeadCare II System

To quantify the concentration of Pb (lead) in the birds’ blood, a 26-gauge surgical needle was first used to puncture the brachial vein. We then used heparinized capillary tubes to collect at least 50 µl of the emerging, 50–150 mL-sized blood droplet. This method of blood collection has been utilized in a variety of avian hematological studies and known to be safer and less invasive than sampling through other methods such as by the jugular vein (Owen 2011). We immediately analyzed each blood sample using a LeadCare II system (Meridian Bioscience) following the manufacturer’s instructions, to generate a measurement of blood-lead concentration (µg/dL). As recommended by the manufacturer, we performed the LeadCare II system’s quality control (QC) procedures each month or upon the opening of a new lot of assay kits-whichever occurred more frequently. Calibration of the system was done internally through the use of a lot-specific key. The QC procedures for each lot of test kits involved assuring that lead concentrations readings generated by the system were within manufacturer-accepted precision ranges for two known reference concentrations of Pb. The lower-bound detection limit was > 3.3 µg/dL and all QC tests were within the procedural limits for the system each time they were performed.

### Comparison of LeadCare II and ICP-MS Analysis of Blood-Pb Concentrations

The LeadCare II system determines blood-Pb concentrations via anodic-stripping voltammetry and, while initially designed for testing human blood, has been increasingly used in wildlife studies (Herring et al. 2018). This is because the system is portable, has a relatively low cost per sample, and is easy to use effectively in field or captive settings (Herring et al. 2018; Zahor 2021; White et al. 2022; Harris 2022). However, the LeadCare II system was designed to analyze Pb concentration in human blood and is known to underestimate blood-Pb concentrations of birds (Zahor 2021; Harris 2022). To account for this suspected bias, we compared LeadCare II results to those generated by inductively coupled plasma mass spectrometry (ICP-MS). ICP-MS offers a more-precise method that while more time-intensive (3–4 weeks to store, ship, and receive analyzed results from an external laboratory) and expensive is highly correlated to precise blood heavy metal analysis methods such as graphite-furnace atomic absorption spectrometry (GFAAS) (Herring et al. 2018).

To generate data for this comparison of LeadCare II and ICP-MS results, we collected a second blood sample from each bird in the main exposure study (see below) after completion of all behavioral trials. Using blood collected from the same birds on the same day, we obtained a LeadCare II measurement (as described above) and sent an additional blood sample of 70 µL in a 1.5-mL Eppendorf tube to an external laboratory for ICP-MS analysis (Dartmouth TEA Lab, Hanover, NH, USA). ICP-MS was performed using an Agilent 8900 Quadrupole instrument and underwent a regular seven-point calibration curve through an internal calibration standard run every 10 samples. For every 20 samples, one blank, one duplicate and one spiked sample was analyzed. The Agilent 8900 machine had a Pb detection limit of samples > 1.107 µg/L and the laboratory followed QC standards put in place by the US Environmental Protection Agency (EPA Method 3050B).

In order to compare these two methodologies, we regressed the LeadCare II measurements and the ICP-MS results of each individual bird using a Pearson correlation. As expected, we found a strong positive association between the LeadCare II and ICP-MS data (linear regression, *r*^*2*^ = 0.944). Using the resulting equation of the line (*y* = 1.57(*x*) + 1.29), we then converted all LeadCare II system estimates of blood-Pb to a concentration predicted by ICP-MS. Consistent with other studies using LeadCare II, our regression analysis showed that LeadCare was underreporting Pb levels by approximately 57%. This represents a value which is in between levels of underreporting other studies have found (Zahor 2021; Harris 2022). While this meant that our pilot study’s Pb blood level levels were slightly higher than our initial predictions, this comparison with ICP-MS aided in better contextualizing the actual Pb load in each of our birds. As the ICP-MS values are better representatives of the birds’ true Pb load, all resulting blood-Pb concentrations reported throughout the rest of this work have been adjusted to reflect this conversion.

### Pilot Study 1 Procedure

To inform the design and exposure levels of the main study, we conducted a 12-week pilot study. We arbitrarily assigned 20 house sparrows (9 female, 11 male) into five groups (*n* = 4 in each) ensuring an even sex ratio in all but one group. We placed each group in a separate aviary with the same conditions as the larger groups in which they had previously been held. We do not think that this change in the social groups of the birds resulted in any behavioral shifts relevant for our individually assessed experiments. Other studies assessing Pb-induced behavioral changes have performed similar changes from large aviaries to treatment groups did not find discernable, aviary-induced differences between treatment groups (Goodchild et al. 2021).

The treatments we exposed these birds to were derived from previous studies on Pb in birds as to create environmentally relevant exposure conditions. We derived five concentrations of Pb acetate and put in each of them in randomly-assigned groups’ drinking water: control (no Pb added to DI water); 100 µg/dL; 200 µg/dL; 400 µg/dL; and 800 µg/dL of Pb/DI water concentration. These concentrations were selected with the intention of reaching ecologically relevant concentrations of Pb in the blood of the birds, using conversion calculations derived from the blood-Pb results reported in Goodchild et al. (2021). For the four Pb treatments, in ascending order, the intended target blood-lead concentrations were: 5 µg/dL (unpublished data from house sparrows caught in Flint, MI, USA, Jamie Cornelius, Dorothy Zahor, Kenneth Glynn), 10 µg/dL (house sparrows caught in urban Vermont-Chandler et al. 2004; approximate of thrushes caught at a polluted site-Zahor 2021), 20 µg/dL (“subclinical poisoning”-Franson and Pain 2011), and 40 µg/dL (average for house sparrows living at a polluted site-Harris 2022), respectively.

To monitor the concentration of Pb in the birds’ blood over time, we collected a blood sample from half of the birds in each treatment aviary once per week. In other words, each bird was bled once every two weeks and two (of the four) birds from each treatment were sampled every week. Due to lack of timely availability of LeadCare II assay kits from the manufacturer during the first 6 weeks of this initial pilot study, we stored collected blood at − 80 °C in EDTA Eppendorf tubes, later defrosted these samples at room temperature, and then immediately analyzed them as described above. All other blood samples were immediately processed on the LeadCare II system without freezing.

Following 12 weeks of exposure to Pb and blood sampling, we ethically euthanized all the Pb-exposed birds. Euthanasia was conducted via rapid decapitation under protocols approved by William and Mary’s IACUC. As to minimize discomfort, birds were held in individual bid bags/ containers where they could not see other birds prior to their own euthanasia. Once the quick procedure was conducted, individuals ceased significant movement and their carcasses were moved to a biohazard container. We kept the four control birds for a second, smaller pilot study to reduce the number of birds taken from the wild.

### Pilot Study 1—Results

The concentration of Pb in the blood of the sparrows during our first pilot study increased during the first 4 weeks of exposure before decreasing slightly over the next 8 weeks and reached an asymptote (Fig. 1a). The final adjusted (following conversion to ICP-MS predictions) average Pb concentrations in each treatment are summarized in Table 1. It should be noted that the LeadCare II system has a lower threshold of detection of 3.3 µg/dL, and all values below that are returned as zero. Hence, this system is not appropriate for assessing very low Pb exposures.

**Table 1:** Summary of treatment and blood-Pb concentrations in the pilot studies

Concentration of Pb in water (µg/dL)	*N*	Average concentration (± SE) of Pb in blood of subjects (µg/dL) at end of study
*Pilot study 1*		
0	4	Undetectable
100	4	Undetectable
200	4	6.28 ± 0.28
400	4	9.18 ± 0.77
800	4	17.23 ± 1.1
*Pilot study 2*		
1600	2	33.72 ± 3.75
3200	2	48.16 ± 0.45

Values are fixed based on reciprocal ICP-MS analysis. “*N*” refers to the sample size, and “SE” represents the standard error of the mean

These blood-Pb concentrations were surprisingly low given our predictions. However, there are very few studies that have exposed birds to Pb from water exposure and fewer in house sparrows. As such, our calculated blood-Pb levels across treatments were based on the results of a study performed with Zebra Finches (*Taeniopygia castanotis*). Zebra finches’ smaller size and unique ecological background in comparison with house sparrows may have influenced the how much treatment water the different species were drinking, the rates of Pb deposition into other tissues beyond blood, or any variety of biological mechanisms related to how Pb interacts within these starkly distinct species (Swaileh and Sansur 2006; Goodchild et al. 2021; Harris 2022). In addition to this more probable reason for our low blood-Pb concentrations, our initial pilots collected blood-Pb levels solely via LeadCare II. As previously described, this method has been known to underreport levels of Pb in avian blood (Zahor 2021; White et al. 2022). At the time of these pilots, we had not done an ICP-MS comparison as to fix our Pb levels, and we under the impression the LeadCare II results could reflect true Pb levels in blood. We did utilize another study’s LeadCare II underestimation value (33% from Zahor 2021) in an attempt to better account for this underreporting (See supplemental materials) but as we found from our ICP-MS, even that value was lower than the true Pb levels in the birds. Therefore, we planned a second pilot study employing higher concentrations of Pb in the DI drinking water to ensure we could reach blood Pb concentrations that were ecologically-relevant yet still sublethal.

### Pilot Study 2—Procedure

We arbitrarily assigned two birds to receive Pb/DI water concentration of 1600 µg/dL and a further two birds to receive 3200 µg/dL in their DI drinking water. We chose these concentrations with the intent of creating blood-Pb concentrations that were representative of highly contaminated environments (Harris 2022) while still being sublethal. The two groups were housed in separate aviaries with the same general housing conditions as before. We monitored birds in pilot study 2 for five weeks (7 October to 9 November 2022) during which we collected blood samples from all birds each week (as described above) and analyzed them on the LeadCare II system to estimate blood-Pb concentrations. At the end of this study, we humanely euthanized the four birds as previously described.

### Pilot Study 2—Results

In our second pilot, birds in both treatments experienced a rapid elevation of blood-Pb concentrations during the first 3 weeks of exposure (Fig. 1b), with a relative asymptote between weeks 4 and 5. The final blood-Pb levels for birds in pilot study 2 are reported in Table 1.

### Main Exposure Study—Procedure

After analyzing the blood-Pb concentrations of birds in both pilot studies, we selected two water-Pb concentrations for the main exposure experiment: a “lower” treatment of 800 µg/dL and a “higher” treatment of 1600 µg/dL. Based on the pilot studies, we predicted that the “lower” treatment would result in a blood-Pb concentration of approximately 17 µg/dL (Table 1), which has been reported as around the average of birds occupying Pb-polluted areas of Flint, MI, USA (Zahor 2021). We predicted the “higher” treatment would result in blood-Pb concentrations of approximately 34 µg/dL which is closer to the limit of sublethal exposure for wild birds (20–50 µg/dL; Franson and Pain 2011).

We conducted the main exposure study between December 2022 and March 2023. All birds utilized in this study experienced approximately 5–6 weeks of acclimation to the aviary before being put into treatment groups. In mid-December 2022, we randomly assigned the 54 wild-caught adult house sparrows (18 female, 36 male) to our three treatment groups (control, lower, higher, *n* = 18 in each treatment) where we maintained a 1:2 sex ratio in each group. Each treatment group was divided into three aviaries (*n* = 6 per aviary; 2 female, 4 male) identical to the general housing described previously. Given how blood-Pb concentrations increased and reached an asymptote over time in the pilot studies (Fig. 1), we exposed birds to Pb for approximately 9 weeks before conducting the following assays.

After 9 weeks of exposure, each bird experienced three behavioral assays in the following order: escape takeoff flight, in-hand struggle assay, and an in-hand breathing rate assay. Birds experienced these assays in the same order over the course of approximately 24 h. Following a further 5–6 days of continued exposure to Pb in housing aviaries, each bird experienced an activity assay.

To report the blood-Pb level of the sparrows closest to the time of all these assays, we collected a blood sample from each bird (as described earlier) for LeadCare II analysis. Specifically, this sampling occurred approximately 48 h after the takeoff flight assay, 24 h after the in-hand struggle and breathing assays, and 4 days before the activity assay. Three weeks after the completion of the activity assay, we collected terminal blood samples to compare blood-Pb concentrations analyzed by LeadCare II and ICP-MS, as described above.

Two birds (1 control, 1 higher treatment) died from unknown causes before they performed behavioral assays. A further bird from the higher treatment experienced a leg injury in the earliest parts of the study and was excluded from escape takeoff and activity assays. Hence, the sample size was reduced in this treatment group.

#### Escape Takeoff Flight Assay

**Fig. 2:**
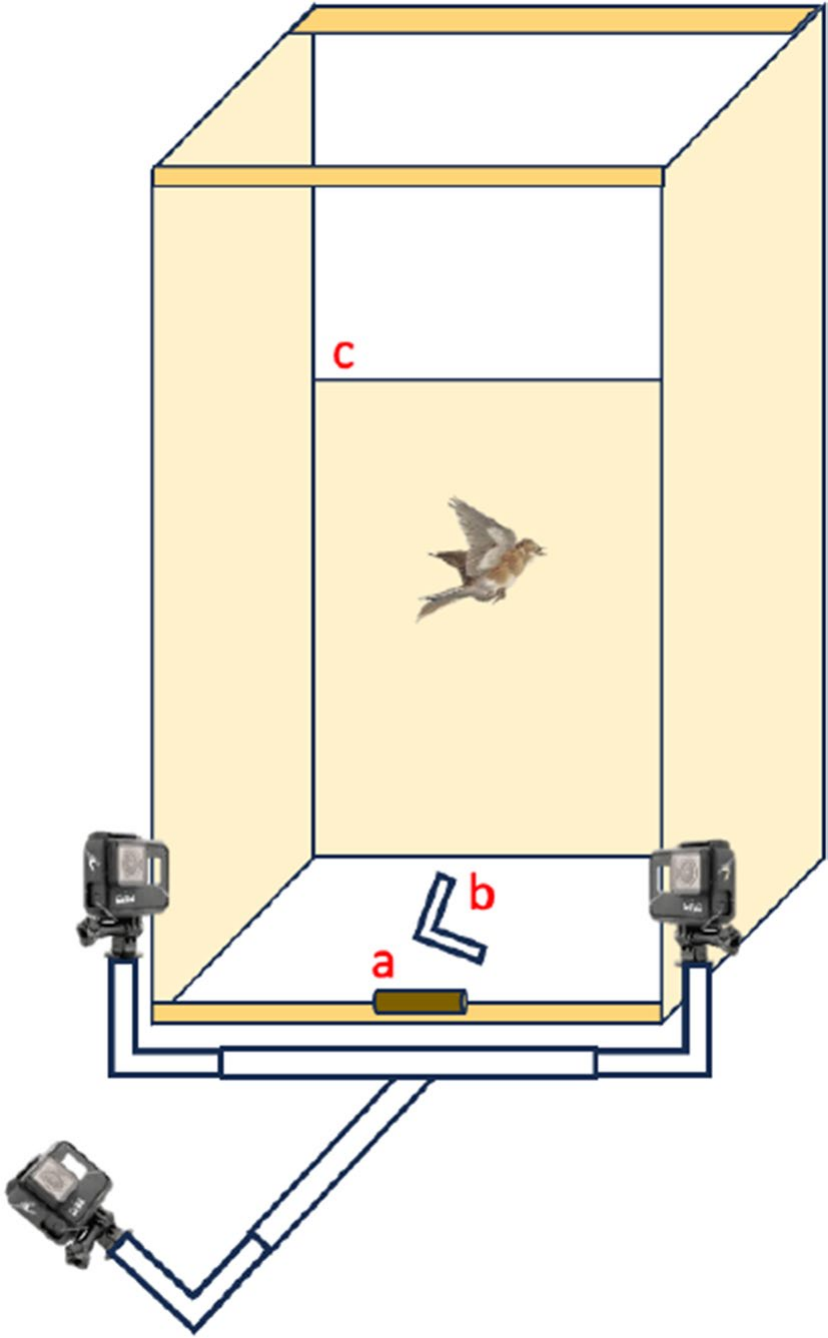
Diagram of escape takeoff flight structure apparatus. The label **a** indicates the wooden release perch; **b** is a small “L”-shaped PVC structure that aided in determining the axes of digitized flights; and **c** signifies the end barrier that birds flew over. Flights were recorded on three fixed GoPro cameras

We conducted escape takeoff flight assays on February 18, 2023, from 11:00 to 15:00 in a long flight aviary (approximately 12 × 9 × 2.5 m) that was adjacent to, but visually separated from the birds’ housing aviaries. Within the flight aviary, we built a structure to encourage the birds to fly in a similar direction upon release (Fig. 2). This structure consisted of two upright wooden boards (180 × 120 cm) on the sides and an upright but shorter wooden barrier (120 × 80 cm) at the end toward which each bird was encouraged to fly. At the release point of the bird, we fixed a 4 cm long wooden dowel to a wooden beam that was at ground level. We also hung a heavy drop-cloth around the structure to further incentivize the bird to fly toward the end barrier and not around the structure. With the experimenter stationed behind the bird and the bird released by hand from the perch and facing toward the end barrier of the structure, each bird flew over the end barrier.

Before a series of flight trials, all birds in an aviary were caught and body mass was taken on a balance to 0.1 g precision (Denver Instrument Co., Model TL-203). An experimenter held a bird in a standard “banders grip” so that its feet grasped the release perch. When the bird was gripping the release perch and in a consistent body position, the experimenter removed their hand from the bird and the bird took off from the perch toward the end barrier of the structure. Following their flight, each bird was caught and placed back in their appropriate housing aviary. In total, 51 birds (control, *n* = 17; lower, *n* = 18; higher, *n* = 16) completed escape takeoff flight trials.

We recorded each takeoff flight on three GoPro Hero7 Black cameras (60 frames per second, 1440 resolution) that were placed in fixed positions to capture flight from different angles (Fig. 2). Before recording a bout of flight trials, we synchronized the video cameras with ambient sound and flashlight cues. We also took measurements to calibrate the air space of the flight structure by digitizing (in Argus software, see below) the positions of two brightly colored polystyrene orbs that were fixed at opposite ends of a 46 cm dowel wand. We recorded paired orb positions throughout the whole air space of the structure. We also recorded on each camera an L-shaped PVC structure that was placed on the ground in the center of the flight structure to determine the spatial orientation of the *x* (left–right), *y* (close-far), and *z* (vertical up-down) axes. The wand was removed before we flew birds in a trial, but the L-shaped structure remained throughout.

We defined the start (frame 1) of a takeoff flight as the first frame where a bird’s feet left the perch as it became airborne. In Argus software (see below), we digitized the approximate centroid of the bird for the first 15 frames of flight. This period accounted for the section of flight where a bird is most likely performing maximally to get off the ground and away from the experimenter (Swaddle et al. 1999; Carlson et al. 2014).

We analyzed video recordings of each flight from each camera in Python version 3.8.3 using the open-source software package, Argus (Jackson et al. 2016). We employed a previously established, well-utilized protocol (Jackson et al. 2016; Emerson et al. 2022; Thady et al. 2022) in which we reconstructed the three-dimensional flight path of each bird. Briefly, the Argus software synchronized the frames from each camera using the sound and light cues that were recorded on each GoPro. It also accounted for the orientation of *x*, *y*, and *z* axes of the flight space and uses the paired calibration points generated by digitization of the orbs to assign real measurements to the volume of air space recorded on the cameras. Following manual digitization of the position of the centroid of each bird on each of the 15 frames of recordings, the software recreates the 3-D position in a calibrated air space so that we can compute metrics of flight performance. For more details about this methodology, see Jackson et al. (2016).

From the 3D coordinates generated in Argus, we computed the energy, in Joules, expended during takeoff (Swaddle et al. 1999). Specifically, we calculated instantaneous velocity by subtracting the value of the previous frame’s (*n *− 1) coordinates from the current (*n*). We multiplied the resultant vector magnitude by 60 (i.e., frame rate of recordings) to yield velocity (*v*) in m/s (Eq. 1; Thady et al. 2022; Emerson et al. 2022).

1 $$v = \sqrt{{({x}_{n}-{x}_{n-1})}^{2}+{(y-{y}_{n-1})}^{2}+{({z}_{n}-{z}_{n-1})}^{2}*60}$$

We used the resulting instantaneous velocity along with the mass of each bird (*m*), the vertical height achieved in each frame (*z*), and the gravity constant (*g*) to calculate both the instantaneous kinetic and potential energy. These were summed to compute the total energy (TE) expended in each frame (Eq. 2; Swaddle et al. 1999). We averaged the energy expended values over the 15 frames of takeoff flight to generate an average energy expenditure over escape takeoff flight.

2 $${\text{TE}} = \frac{1}{2}\left[(m*{v}^{2})\right]+[\left(m*z*g\right)]$$

We also calculated the initial takeoff leap force (TOF) to quantify the force generated by the bird’s legs over the first two frames of takeoff (Provini et al. 2012; Bosner and Rayner 1996). This was calculated by multiplying the acceleration of the bird in that second frame (second-frame velocity, or sfv^*2*^*,* in m/s^2^) by each birds’ mass (*m*, in kg) (Eq. 3).

3 $${\text{TOF}}={{\text{sfv}}}^{2}*m$$

#### In-hand Struggle Assay

We selected birds at random (*n* = 52) for in-hand struggle assays, between 10:00 and 16:00 on February 19, 2023. Following capture with a hand net, each bird was placed in a cloth, opaque bird bag for 2 min. Using methods adapted from Brommer and Kluen (2012), a single experimenter took a bird from the bag and placed it in standard banders’ grip using similar pressure for all birds. The experimenter was blind to the treatment group. The bird was held upright approximately 30 cm from the experimenter’s face and the experimenter counted the number of physical struggles (defined as movements of the body that could be detected by the experimenter) exhibited by the bird in a 30 s period.

#### In-hand Breathing Rate Assay

Immediately following the struggle assay, the bird was held in a horizontal position on its back closer to the experimenter’s face (approximately 15 cm) with its legs fully secured by the experimenters’ second hand to expose the bird’s chest. This allowed the experimenter to observe movements of the chest associated with breathing (Carere and van Oers 2004). The experimenter counted these movements for 30 s, to estimate a breathing rate. Immediately following the first 30 s bout, the assay was repeated to generate a second estimate of breathing rate. We averaged these two estimates to generate a single breathing rate for each individual bird. Following the breathing rate assay, each bird was returned to its housing aviary.

#### Activity Assay

**Fig. 3:**
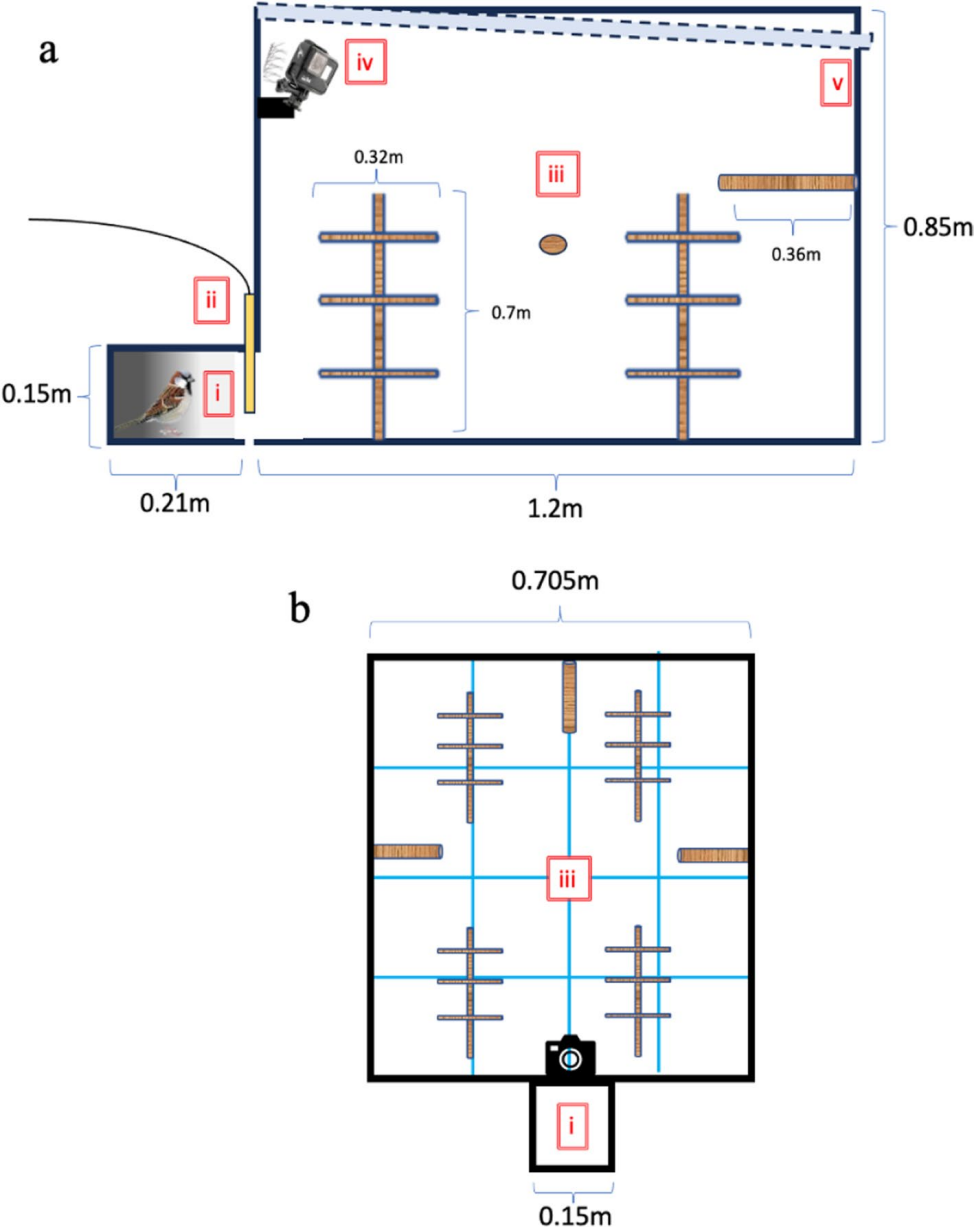
Side view (**a**) and plan view (**b**) of the novel environment used in activity assays. The labels indicate the following: (i) darkened release chamber; (ii) release chamber door; (iii) well-lit novel environment with grid pattern on floor; (iv) GoPro camera position; and (v) translucent roof

On February 25–26, five or six days after the previous assays, we quantified metrics of locomotor activity by utilizing a novel environment test (Fig. 3). Similar methods have been used to infer aspects of avian locomotory behavior such as exploration (McCowan et al. 2015; Huang et al. 2012), risk-taking (Pobleté et al. 2021), and boldness (McCowan et al. 2015). We performed each activity assay between 09:00 and 15:00 in a soundproof, indoor aviary (3L × 3W × 2.5H m). Though birds were tested individually, we tested all individuals from the same treatment aviary sequentially. The order of treatment aviaries was randomized.

The activity arena comprised two sections: a small, dark release chamber (20L × 15W × 15H cm) and a larger activity chamber (120L × 70.5W × 85H cm) (Fig. 3). These two chambers were aligned so that when a door was opened, a focal bird could move from the release chamber to the activity chamber. The activity arena was constructed with plywood walls and floor and a translucent ceiling (Ejoy, 121.92L × 60.96W × 0.78 cm thickness, corrugated poly-plastic roofing panel) that allowed the ambient light of the room to illuminate the activity chamber. We placed four perching structures (each with a single vertical post 69 cm tall and three horizontal perches that were 35 cm long, each constructed from 1.5 cm diameter bamboo) in the chamber. In addition, we attached an additional wooden-dowel perch (36 cm long, 2 cm diameter) to the walls of the activity chamber as shown in Fig. 3. We also attached a GoPro (Hero7 Black, 60 fps, 1440 resolution) to the wall closest to the entrance of the activity chamber. Though we placed hardware wire deterrent above the camera, some birds tried to perch in that blind spot (13 attempts by 8 individuals); hence, we counted the camera location as another perch. In total, there were 16 separate perch locations (12 attached to free-standing vertical posts, three attached to the chamber walls, and the GoPro camera). We marked the floor of the activity chamber with a grid pattern (16 rectangles, 30 × 18 cm) so we could note which part of the chamber a bird occupied. Birds could be visualized on all perches and in each area during the whole trial.

To begin an activity trial, we placed a focal bird into the release chamber for 5 min (Fig. 3) (McCowan et al. 2015). We then opened the door to the activity chamber, and in most cases (*n* = 40 of 51), the bird entered the activity chamber immediately. In the 11 cases when this did not happen, the experimenter waited 10 s and tapped on the outside of the release chamber to encourage the bird to enter the activity chamber. All 11 birds entered the activity chamber with that additional stimulation. We video recorded all movements and behaviors of the focal bird for 8 min. Following each activity trial, the focal bird was returned to its housing aviary. We cleaned the activity chamber between trials to minimize cues left from the previous bird.

From the 8-min videos (*n* = 51), we quantified the following five behaviors. (1) “Flights,” defined as the total number of movements of the focal bird that involved use of its wings and/or movements between perches. (2) “Hops,” defined as the total number of leg-based bouts of movement on the floor of the chamber or along a single perch. (3) “Perches visited,” defined as the total number of unique perches (max = 16) that a bird visited. (4) “Areas visited,” defined as the total number of unique grid zones (max = 16) the bird visited. (5) “Self-maintenance behaviors,” defined as the total number of bouts of self-maintenance activity, including preening and bill wiping.

### Main Exposure Study—Statistical Analyses

To assess the effect of Pb exposure on escape takeoff flight, we compared both average energy expended during takeoff and takeoff force across the three treatments (control, lower, higher) with separate one-way ANOVAs. We employed Tukey’s post hoc test to examine differences between our individual treatments. To ensure our results met the normality assumption for using an ANOVA, we conducted a Shapiro–Wilks test on extracted residuals.

Similarly, we examined among-treatment differences in the in-hand breathing rate metric with a one-way ANOVA and outcomes from the in-hand struggle assay with a Kruskal–Wallis rank sum tests as the residuals from this latter analysis were non-normally distributed.

We reduced the dimensionality of the five behavioral metrics of the activity assay using principal components analysis (PCA). The PCA rendered two interpretable principal components (see Results for descriptions). We compared among-treatment differences in PC1 and PC2 using separate Kruskal–Wallis rank sum tests. If statistically supported, we performed the post hoc Dunn tests to tease apart differences between treatments.

All analyses were performed and visualized in R version 4.2.1 (R Development Core Team, 2021) interpreting two-tailed tests of probability.

## Results—Main Exposure Study

Blood samples taken 9 weeks after exposure, or approximately at the time of our assays, showed that birds in the lower treatment group had an average (± standard error) blood-Pb concentration of 16.68 (± 3.93) µg/dL. Birds in the higher treatment had an average of 30.04 (± 7.08) µg/dL. Two of the 18 control birds had detectable Pb in their blood (6.64 µg/dL and 6.80 µg/dL). Due to these being wild-caught birds from urban areas, it is possible that they had incurred this minor exposure before coming into captivity; both were in separate control aviaries and all other individuals within each aviary had undetectable levels of blood-Pb despite drinking from the same dispenser. Besides these two individuals, all other control birds had blood-Pb levels that were well below the detectable level of our LeadCare II system.

**Fig. 4:**
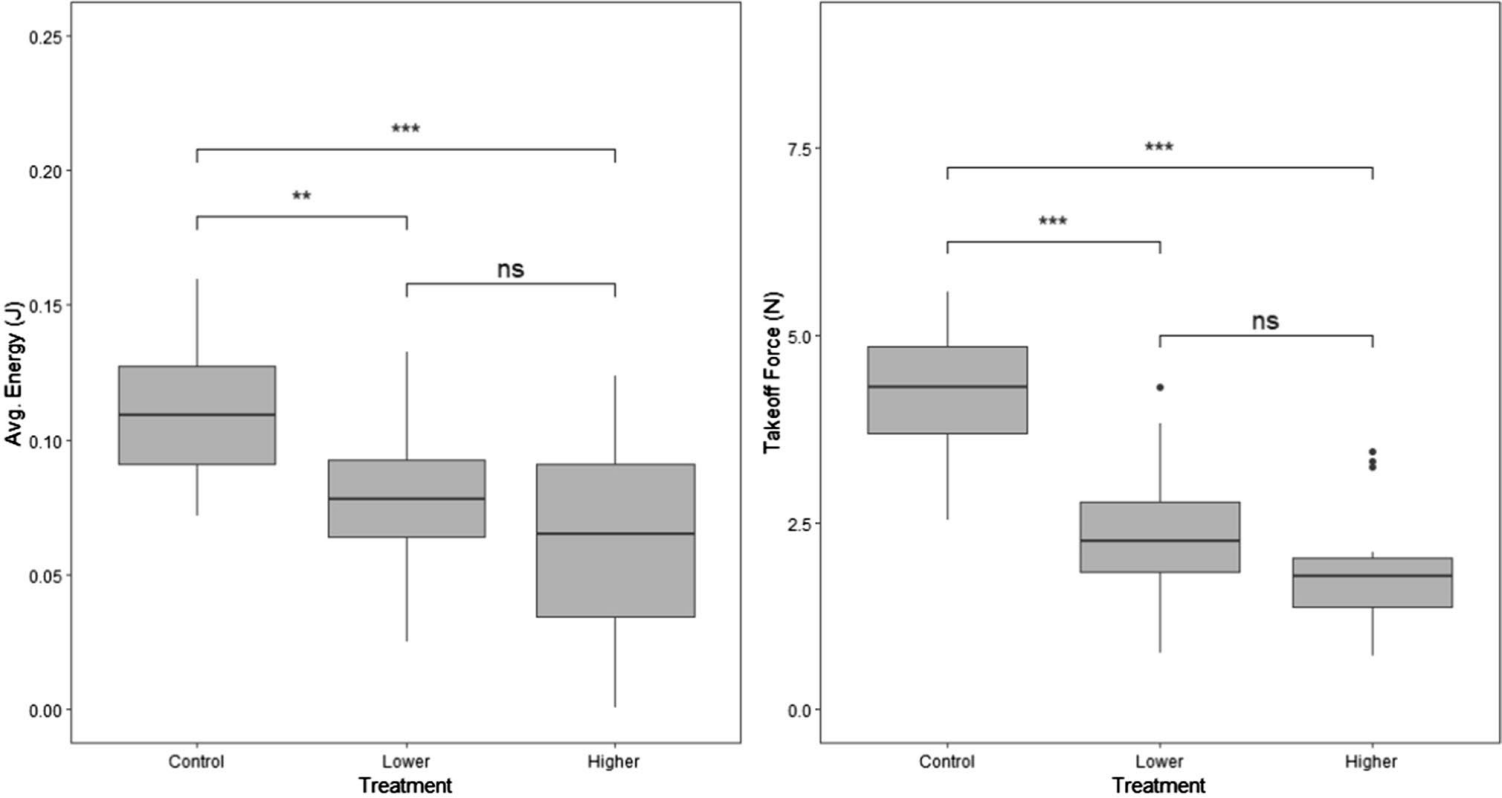
Boxplots of **a** average energy expended during takeoff and **b** takeoff leap force by Pb treatment (blue = control, yellow = lower, red = higher). The black bar inside the boxplot represents the median of data, the limits of the boxes represent the inter-quartile range, and the lines extending from the boxes represent the extremities of the data. Black dots are outliers of the data. ** indicates a statistical comparison where *p* < 0.001 and *** indicates *p* < 0.0001 level; “ns” represents a comparison in which *p* > 0.05

Exposure to Pb significantly influenced energy expended during takeoff flight (Fig. 4a; *F*_2,48_ = 9.71, *p* = 0.0003). Birds in both the lower (Tukey HSD, *q* = − 0.034, *p* = 0.007) and higher (*q* = − 0.047, *p* = 0.0003) Pb treatments expended less energy than controls. However, we did not detect differences between the lower treatment and higher treatment (*q* = − 0.013, *p* = 0.487). Similarly, Pb treatment influenced takeoff leap force (Fig. 4b; *F*_2,48_ = 33.64, *p* < 0.000001) such that birds in both lower (*q* = − 1.90, *p* < 0.0001) and higher (*q* = − 2.34, *p* < 0.0001) Pb treatments exerted less force in their takeoff leap than controls. There was no difference in takeoff leap force between birds in lower and higher Pb treatments (*q* = − 0.435, *p* = 0.109).

Exposure to Pb did not influence the number of struggles observed in a 30 s handling period (*χ*^2^ = 0.808, *p* = 0.668). The results of the two individual breathing rate assays were highly correlated (*r* = 0.938); however, the resulting average breathing rate while being handled (*F*_2,49_ = 0.564, *p* = 0.573) did not vary between Pb treatment groups and/or control.

The PCA of the five activity metrics recorded in the novel environment returned two PCs, which explained 87.5% of the original variation (Table 2). PC1, which we interpreted as total “movement activity,” explained 69% of the variation and was positively loaded with all five behaviors. PC2, which we interpreted as “Movement-based lack of self-maintenance,” explained an additional 18.5% of the variation and was positively loaded with “areas visited” and “hops” and negatively loaded with “self-maintenance.”

**Table 2:** Loading factors for PC1 (principal component 1) and PC2 (principal component 2) from the principal component analysis of activity metrics in the novel environment

Behavior/variable	PC1 (69% of variation)	PC2 (18.5% of variation)
Total flights	0.899	− 0.267
Total hops	0.910	0.333
Perches visited	0.864	− 0.120
Areas visited	0.763	0.604
Total self-maintenance	0.697	− 0.603

**Fig. 5:**
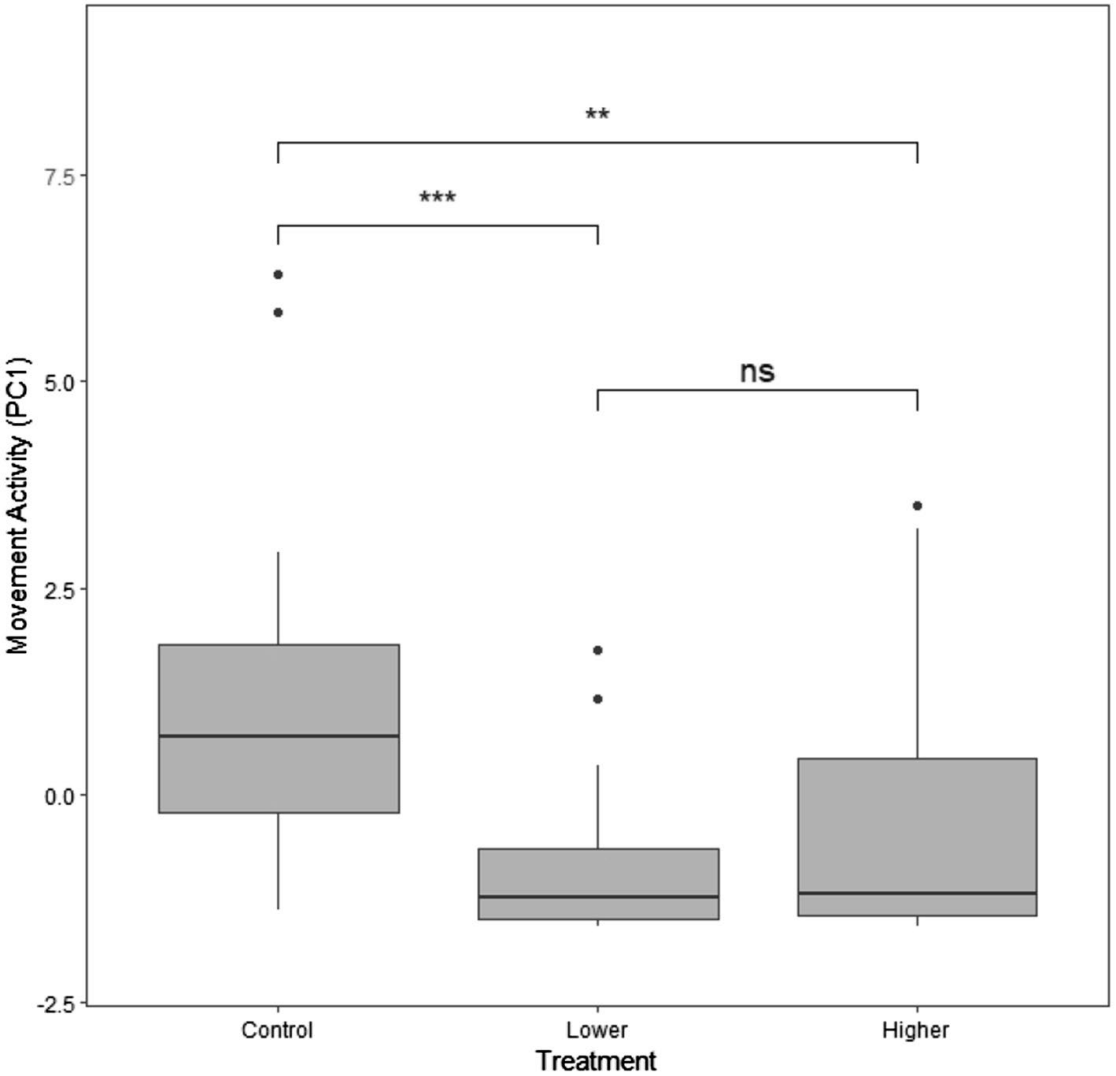
Boxplot of PC1 (movement) activity by Pb treatment (blue = control, yellow = lower, red = higher). The black bar inside the boxplot represents the median of data, the limits of the boxes represent the inter-quartile range, and the lines extending from the boxes represent the extremities of the data. Black dots are outliers of the data. ** indicates a statistical comparison where *p* < 0.001 and *** indicates *p* < 0.0001 level; “ns” represents a comparison in which *p* > 0.05

Exposure to Pb influenced PC1 (movement activity) scores (Fig. 5; *χ*^2^ = 12.6, d*f* = 2, *p* = 0.0018) but not PC2 (movement-based lack of self-maintenance) scores of birds (*χ*^2^ = 0.120, d*f* = 2, *p* = 0.942). Within the PC1 results, birds from both lower (*z* = 3.44, *p* = 0.0009) and higher (*z* = 2.47, *p* = 0.021) Pb treatments had lower PC1 (movement) scores than controls. There was no difference in PC1 scores between birds in lower and higher Pb treatments (*z* = 0.889, *p* = 0.561).

## Discussion

House sparrows exposed to a sublethal concentration of Pb in their drinking water exhibited decreased performance in our biomechanically related behavior assays of takeoff flight and movement activity. In takeoff, birds that were exposed to Pb exerted less force in their initial leap from the perch and expended less energy in flight from the ground. In the movement assay, Pb-exposed birds were less active. While both of our Pb treatments differed from controls, we did not see differences between our lower and higher Pb treatment groups. This is consistent with the lower Pb treatment being above a threshold that induces decreases in biomechanical performance.

It is likely that the performance reductions in both behaviors are indicators of some combination of Pb-induced neurological and muscular impairment. When compared to our controls, birds in our lower Pb treatment group exhibited a 17% decrease in average energy expended across the start of takeoff flight. Similarly, birds in our higher Pb treatment group exhibited a 27% decrease in energy during the start of their flight. In takeoff flight, the majority of biomechanical forces are generated by the pectoralis muscles of birds (Swaddle et al. 1999). Meanwhile, in the initial leap from the perch, the power is largely generated by hindlimb muscles (Bonser and Rayner 1996). We observed similar magnitudes of reduced performance in this takeoff leap compared with takeoff flight. Birds in the lower Pb treatment exerted 45% less force and birds in the higher Pb treatment 55% less force than control birds. Consistent with decreases in takeoff performance, overall movement activity was reduced in birds exposed to sublethal levels of Pb. Birds in the lower treatment group showed an averaged 69% decrease across the five behaviors we examined (total numbers of flights, hops, areas visited, perches visited, and self-maintenance behaviors). Meanwhile, birds in the higher treatment groups experienced an average decrease of 52% across these behaviors. Interpreting the takeoff and movement data together, we conclude that exposure to sublethal Pb leads to substantially decreased biomechanical performance of several major muscle groups in the avian body and is consistent with Pb’s predicted effect on the physiological systems involved in these responses.

The physiological pathways through which Pb exposure impacts biomechanical function remain largely unknown. This spectrum of effects may encompass various physiological and skeletal-muscular mechanisms that have been shown to shape the avian behaviors we studied (Swaddle and Witter 1997; Provini et al. 2012; Careau et al. 2008).

Proper hematological responses play a crucial role in birds’ abilities to regulate oxygen around their body (Butler 2016); hence, Pb-induced impairment in hematological responses may compromise oxygen-intensive behaviors such as flight and other related movements. Multiple measures of avian blood health are known to be negatively impacted by Pb’s oxidative properties such as the inhibition of δ-aminolevulinic acid dehydratase (ALAD), an enzyme involved with the heme-biosynthetic pathway (Cid et al. 2018). Due to ALADs role in blood synthesis, inhibition of this enzyme has been closely related to poor body condition and anemia in birds (Roux and Marra 2007; Berglund et al*.* 2010; Sato et al. 2022). Birds experiencing these physiological conditions would likely suffer muscular or other organ impairments which could explain the decreases in behavior we observed. Other indicators of hematological health are also known to be impacted by exposure to Pb, such as erythrocyte cell volume (Beyer et al*.* 1988), plasma protein concentration (Vallverdú-Col et al*.* 2016), and hemoglobin levels (Beyer et al*.* 1988). With less efficient oxygen transport, blood health, and blood synthesis, biomechanical behaviors like flight and movement would likely suffer analogous decreases.

Other physiological systems that contribute to our studied behavior may have been affected by Pb exposure. It is possible that fundamental properties of the avian skeleton could be compromised by exposure to Pb, as the heavy metal competes with calcium during bone strengthening and repair (Ishii et al. 2018). Since bone structure is a key component of both birds’ oxygen regulation and flight ability (Butler 2016), increased Pb uptake by skeletal systems could make movements and powered flight less efficient. Also, sublethal Pb has also been known to disrupt processes related to the endocrine system, specifically those related the stress-related HPA axis (Baos et al. 2006; White et al. 2022; Zheng et al. 2022). As our assays all involved some degree of handling by a human threat, improper or decreased stress responses may have contributed to the behavioral reductions in flight and activity observed.

In addition to physiological impairments, exposure to Pb may also interfere with neurological functions associated with the behaviors we tested. In vertebrates, it is well-known that the oxidative properties of Pb often make the brain and other parts of the central nervous system targets of Pb accumulation (Douglas-Stroebel et al. 2005; Nam et al. 2012; Cid et al. 2018). When present, exposure to Pb has been associated with increased inflammatory responses, histopathological damage, demyelization, and apoptosis of neurons in vertebrates (Neal and Guilarte 2010; Hossain et al. 2016; Zheng et al. 2022). Most prominently, Pb’s negative impact on the vertebrate nervous system has been well-studied at neural synapses where it causes damage by competing with calcium ions (Ca^2+^) resulting in pre-synaptic and post-synaptic losses in neurological function (Neal and Guilarte 2010; Zou et al. 2020).

Pb’s deleterious impacts on the functioning of the neural synapse is well-known. Pre-synaptically, chronic exposure to Pb’s oxidative properties results in impaired neurotransmission through the reduction of Ca^2+^-dependent glutamate and y-aminobutyric acid (GABA) release (Nam et al. 2012; Cid et al. 2018; Zou et al. 2020). The decrease in these neurotransmitters within the synapse has been related to the decreased function of multiple parts of the avian neurological system including the hippocampus-a brain region vital for the retention of spatial information, visual task success, and memory (Colombo and Broadbent 2000). Inaccurate processing of spatial information may have impaired the proper control of movement in our birds, resulting in the decreased behavioral endpoints we report.

In addition to disrupting neurotransmitter release, Pb exposure has been linked to post-synaptic decreases in neural function. For example, heightened Pb acts as an inhibitor in pathways associated with N-methyl-D-aspartate (NMDAR)-receptor retrograde signaling (Neal and Guilarte 2010). This neurological pathway is key in the utilization and regulation of brain-derived neurotrophic factor (BDNF). BDNF is known to play an important role in a variety of neurological and gene-regulating processes that modulate activation pathways crucial for proper neuronal growth, survival, and synaptogenesis across multiple parts of the vertebrate brain (Colombo and Broadbent 2000; Neal and Guilarte 2010). As a result, sublethal Pb exposure may have reduced aspects of this pathway augmenting the amount of BDNF certain neurons received. This may have resulted in less abundant, further synapses within brain regions associated with motor control such as the cerebellum. Any of these non-mutually exclusive, neurological processes may have been affected by Pb exposure and could have resulted in the decreased biomechanical and motor performance we observed in our sparrows.

Whichever mechanisms underly the effects, the Pb-induced reductions in takeoff flight performance we observed are likely to influence the complex yet crucial ability of birds to escape from predators (Fernández-Juricic et al. 2006). In the case of a songbird evading a ground-based predator, the start of takeoff from the ground is the most crucial part of flight (Veasey et al. 2001). Therefore, individuals moving more slowly and gaining less height, as we observed, are less likely to evade predation (Witter et al. 1994; Swaddle et al. 1999). Apart from anti-predator evasion, decreased flight performance may have detrimental consequences for other avian activities such as seasonal migration (Seewagen 2020), food provisioning (Crino et al. 2017), reproductive output (Byers et al. 2010), and resource competition (Ecke et al. 2017). Therefore, Pb-induced decreases in flight performance may result in a suite of outcomes that likely lower avian fitness.

Similarly, decreases in overall movement activity due to Pb exposure may result in a variety of context-dependent effects. Activities associated with movement are associated with many aspects of avian behavior. Birds not actively moving around their environment, particularly a novel one, may be less likely to find conspecifics, avoid predators, find shelter, and exploit unfamiliar resources such as food (Huang et al. 2012). Furthermore, a lack of spatial movement could also reduce a bird’s ability to gather information on their local environment. Even considering many birds’ wide field of view, movement is commonly necessary to properly sense as well as gain information on their surroundings (Fernandez-Juricic 2012). While there are some indirect metabolic and physiological costs associated with engaging in movement-intensive behaviors (Careau et al. 2008), larger opportunity costs associated with not exploiting resources and perceiving changes in their environment can be major drivers of survival for birds.

The combined negative impact of Pb exposure on birds’ takeoff flight and movement behaviors would likely have ecological consequences in wild populations. The observed decreases in takeoff flight performance indicate that Pb-exposed birds are likely more susceptible to depredation. While this has implications for individual fitness, it also may result in more opportunities for Pb to bioaccumulate in predatory species and subsequently be biomagnified up the food chain, leading to subsequent damage to these higher-trophic level organisms (Abbasi et al. 2015; Einoder et al*.* 2018). However, the ecological effects of Pb exposure are not solely limited to increasing the risk of heightened Pb exposure in upper trophic level organisms. The observed decreases in both takeoff flight performance and general activity levels suggest that birds under the influence of sublethal Pb would struggle with the multitude of behaviors that rely on the related neuro-muscular processes. For example, impaired predatory birds may find it more difficult to hunt efficiently, and as a result, there could be altered predation pressures on their prey species. Similarly, prey species with decreased neurological function may experience memory or movement deficiencies decreasing foraging success and safety.

If exposure to Pb, of the order we studied here, imposes fitness effects in populations, then we would expect to see longer-term evolutionary responses in species such as the house sparrow that tend to be sedentary and exist at high population numbers. Selection for heritable traits that increase resistance to toxicity may provide benefits. In the highly contaminated Restronguet Creek of southwestern England, the polychaete *Hediste diversicolor* exhibited a heritable tolerance to copper (Cu) and zinc (Zn) in a number of field studies (Bryan and Hummerstone 1973, Grant et al. 1989). The mechanistic basis for its relatively low Zn levels appears to be the rate of internal Zn storage which matches the high rate of uptake, preventing toxin-incurred damages. On the other hand, Cu concentrations were elevated, but were mostly in detoxified, insoluble forms (Mouneyrac et al. 2003). Similarly, damage from sublethal Pb exposure could likewise be mitigated by the upregulation of pre-existing detoxifying mechanisms.

However, it is possible that the over-selection of traits that confer such strong resistance to an anthropogenic stressor like Pb may incur maladaptive tradeoffs. Genetic adaptations which facilitate tolerance often involve an organism reallocating energy away from growth and toward detoxification and damage repair (Mouneyrac et al. 2011). One study in lineages of least killifish (*Heterandria formosa*) with laboratory-induced resistances to cadmium (Cd) found a significant tradeoff between Cd tolerance and reproductive fitness. Members of the resistant group had 18% reduced fecundity by the seventh filial generation (Xie and Klerks 2004). Additionally, contaminant-tolerant populations may be more sensitive to future disturbances or less competitive in uncontaminated habitats due to weakened genetic variation, smaller body size, and reduced tolerance to other toxicants (Calow 1991, Mouneyrac et al. 2011). The adaptation to Cu and Zn in species such as *H. diversicolor* is thought to be responsible for its competitive inferiority beneath a threshold concentration of each metal in sediment (Grant et al. 1989).

We predict patterns over time that are consistent with selection for heritable traits that confer greater resistance or tolerance of Pb toxicity (Klerks and Weis 1987). Interestingly, a recent study reported allele frequency differences between two lead contaminated towns in Australia compared to control towns, at loci that have been linked to metal ion transport and lead pollution in other organisms, suggesting that these populations were adapting to contamination (Andrew et al. 2019). While there is evidence supporting species' persistence in adapting to Pb-polluted conditions, the long-term effects of this adaptation are less clear.

We did not find any reliable evidence that exposure to Pb influences the in-hand struggle or breathing rate assays. This could mean that the birds’ assessments of risk imposed by handling are little affected by Pb, but it is also likely that the prior handling and longer-term captive housing of the house sparrows made them generally less reactive to these handling-based assays. They were likely somewhat habituated to the presence and activity of people and these two assays may not be as revealing in such captive situations as they are when measured in free-living subjects. These assays were originally developed and implemented in a study with wild-caught birds that were not acclimated to handling or captive housing. As a result, future studies looking to assess struggling or aggressive behaviors in captive house sparrows should employ methodologies that could be less directly impacted by captivity or habituation to experimenters.

Both of our treatments resulted in sparrow blood-Pb levels that are ecologically relevant. The induced concentrations of Pb observed in the house sparrow’s blood from our lower treatment (average = 16.67 µg/dL) are reasonably reflective of those found in wild birds in polluted environments. For example, American robins (*Turdus migratorius)* living in urban areas contaminated by Pb-polluted water runoff in Flint, MI, USA, during the ongoing Flint Water Crisis were found to exhibit an average of 14.1 and a maximum of 32.0 µg/dL blood-Pb (Zahor 2021). In addition, several populations of great tits (*Parus major*) living in polluted areas in central Europe displayed blood-Pb concentrations within the range of about 10–28 µg/dL (Geens et al. 2010; Bauerová et al. 2020), as did mallards (*Anas platyrhynchos*) in polluted areas in Poland (Binkowski and Meissner 2013). There is also some precedent for the blood-Pb concentrations observed in our higher treatment (average = 30.04 µg/dL) to be found in free-living bird populations. For example, pied flycatchers (*Ficedula hypoleuca*) that lived close to an ore smelter in Finland reported blood-Pb concentrations of up to 42 µg/dL (Berglund et al. 2010). Similarly, resident house sparrows living close to an ore smelting facility in Broken Hill, Australia, were observed to have an average blood-Pb concentration of 39.8 µg/dL (Harris 2022). While relatively uncommon, we posit that our higher treatment level is useful for interpreting the effects of more extreme contamination events in nature. It seems likely that birds exposed to this concentration of Pb experience “subclinical poisoning,” which refers to a level of Pb exposure where physiological effects are reported despite not obviously influencing mortality (Franson and Pain 2011).

It is worth noting the limitations of this experimental work. This study was conducted in a controlled captive environment and therefore cannot completely replicate the process of lead exposure and its consequences as it occurs in a bird’s natural environment. We employed a method of continuous Pb dosing that, while potentially reflective of some populations of wild birds, cannot encapsulate the full range of scenarios through which birds are exposed to Pb. Additionally, although house sparrows can serve as a model for passerine species, it is important to note variation between species with respect to behavior and sensitivity to metal pollution. The house sparrow is particularly useful for studies modeling Pb pollution in urban settings, but may not be the most accurate representative of all other songbirds, particularly those with non-sedentary life histories (Swaileh and Sansur 2006).

Future studies can utilize these results to fill remaining knowledge gaps. Given the wide array of songbirds and their diverse life histories and physiologies, it would be useful to conduct a multi-species comparison of behavior. This would allow for the more complete examination of Pb pollution’s effects across different species and ecological roles. Additionally, future work studying the impact of sublethal Pb’s effect on behavior should center on other behaviors reliant on unimpeded flight, movement or other biomechanical processes, such as song generation, reproduction, or foraging. Finally, an investigation into how these relevant levels of Pb impact the functioning of neurons specifically associated with motor control and function would be ideal as to build a better mechanistic understanding of the behavioral trends we observe.

In summary, takeoff flight and movement activity, two behavioral metrics that likely influence fitness outcomes of birds, were impaired when captive house sparrows were experimentally exposed to sublethal concentrations of Pb representative of contaminated sites. We have yet to identify the mechanisms by which Pb affects these biomechanical and movement metrics, though it is likely that a suite of physiological and neurological processes are disrupted by the exposure to this heavy metal. The decreases in behavioral performance that we report in this study are likely indicative of what populations of wild birds inhabiting Pb-polluted areas may experience, potentially affecting the evolutionary trajectories of exposed individuals, as well as the functioning of the surrounding ecological community.

### Supplementary Information

Below is the link to the electronic supplementary material.

Supplementary file1 (XLSX 16 KB)

## Data Availability

The datasets and *R* code generated during and/or analyzed in the current study are available from the corresponding author on reasonable request.

## References

[CR1] Abbasi NA, Jaspers VLB, Chaudhry MJI, Ali S, Malik RN (2015). Influence of taxa, trophic level, and location on bioaccumulation of toxic metals in bird’s feathers: a preliminary biomonitoring study using multiple bird species from Pakistan. Chemosphere.

[CR2] Andrew SC, Taylor MP, Lundregan S, Lien S, Jensen H, Griffith SC (2019). Signs of adaptation to trace metal contamination in a common urban bird. Sci Total Environ.

[CR3] Assi MA, Hezmee MNM, Haron AW, Sabri MYM, Rajion MA (2016). The detrimental effects of lead on human and animal health. Vet World.

[CR4] Baos R, Blas J, Bortolotti GR, Marchant TA, Hiraldo F (2006). Adrenocortical response to stress and thyroid hormone status in free-living nestling white storks (*Ciconia ciconia*) exposed to heavy metal and arsenic contamination. Environ Health Perspect.

[CR5] Bauerová P, Krajzingrová T, Těšický M, Velová H, Hraníček J, Musil S, Svobodová J, Albrecht T, Vinkler M (2020). Longitudinally monitored lifetime changes in blood heavy metal concentrations and their health effects in urban birds. Sci Total Environ.

[CR6] Berglund ÅMM, Ingvarsson PK, Danielsson H, Nyholm NEI (2010). Lead exposure and biological effects in pied flycatchers (*Ficedula hypoleuca*) before and after the closure of a lead mine in northern Sweden. Environ Pollut.

[CR7] Beyer WN, Spann JW, Sileo L, Franson JC (1988). Lead poisoning in six captive avian species. Arch Environ Contam Toxicol.

[CR8] Binkowski ŁJ, Meissner W (2013). Levels of metals in blood samples from Mallards (*Anas platyrhynchos*) from urban areas in Poland. Environ Pollut.

[CR9] Bonser RHC, Rayner JMV (1996). Measuring leg thrust forces in the common starling. J Exp Biol.

[CR10] Brommer JE, Kluen E (2012). Exploring the genetics of nestling personality traits in a wild passerine bird: testing the phenotypic gambit. Ecol Evol.

[CR11] Bryan GW, Hummerstone LG (1973). Adaptation of the polychaete *Nereis diversicolor* to estuarine sediments containing high concentrations of zinc and cadmium. J Mar Biol Assoc UK.

[CR12] Burger J, Gochfeld M (2000). Effects of lead on birds (*Laridae*): a review of laboratory and field studies. J Toxicol Environ Health-B Crit Rev.

[CR13] Butler PJ (2016). The physiological basis of bird flight. Philos Trans R Soc B Biol Sci.

[CR14] Byers J, Hebets E, Podos J (2010). Female mate choice based upon male motor performance. Anim Behav.

[CR15] Calow P (1991). Physiological costs of combating chemical toxicants: ecological implications. Comp Biochem Physiol.

[CR16] Careau V, Thomas D, Humphries MM, Réale D (2008). Energy metabolism and animal personality. Oikos.

[CR17] Carere C, Van Oers K (2004). Shy and bold great tits (*Parus major*): body temperature and breath rate in response to handling stress. Physiol Behav.

[CR18] Carlson JR, Cristol DA, Swaddle JP (2014). Dietary mercury exposure causes decreased escape takeoff flight performance and increased molt rate in European starlings (*Sturnus vulgaris*). Ecotoxicology.

[CR19] Chandler RB, Strong AM, Kaufman CC (2004). Elevated lead levels in urban house sparrows: a threat to sharp-shinned Hawks and Merlins?. J Raptor Res.

[CR20] Chen TH, Gross JA, Karasov WH (2006). Sublethal effects of lead on northern leopard frog (*Rana pipiens*) tadpoles. Environ Toxicol Chem Int J.

[CR21] Cheng H, Hu Y (2010). Lead (Pb) isotopic fingerprinting and its applications in lead pollution studies in China: a review. Environ Pollut.

[CR22] Cid FD, Fernández NC, Pérez-Chaca MV, Pardo R, Caviedes-Vidal E, Chediack JG (2018). House sparrow biomarkers as lead pollution bioindicators: evaluation of dose and exposition length on hematological and oxidative stress parameters. Ecotoxicol Environ Saf.

[CR25] Colombo M, Broadbent N (2000). Is the avian hippocampus a functional homologue of the mammalian hippocampus?. Neurosci Biobehav Rev.

[CR26] Crino OL, Klaassen van Oorschot B, Crandell K, Breuner CW, Tobalske BW (2017). Flight performance in the altricial zebra finch: developmental effects and reproductive consequences. Ecol Evol.

[CR27] Douglas-Stroebel EK, Brewer GL, Hoffman DJ (2005). Effects of lead-contaminated sediment and nutrition on mallard duckling behavior and growth. J Toxicol Environ Health A.

[CR28] Ecke F, Singh NJ, Arnemo JM, Bignert A, Helander B, Berglund ÅMM, Borg H, Bröjer C, Holm K, Lanzone M, Miller T, Nordström Å, Räikkönen J, Rodushkin I, Ågren E, Hörnfeldt B (2017). Sublethal lead exposure alters movement behavior in free-ranging golden eagles. Environ Sci Technol.

[CR29] Einoder LD, MacLeod CK, Coughanowr C (2018). Metal and isotope analysis of bird feathers in a contaminated estuary reveals bioaccumulation, biomagnification, and potential toxic effects. Arch Environ Contam Toxicol.

[CR30] Emerson LC, Thady RG, Robertson BA, Swaddle JP (2022). Do lighting conditions influence bird-window collisions?. Avian Conserv Ecol.

[CR31] Fernández-Juricic E (2012). Sensory basis of vigilance behavior in birds: synthesis and future prospects. Behav Proc.

[CR32] Fernández-Juricic E, Blumstein DT, Abrica G, Manriquez L, Bandy Adams L, Adams R, Daneshrad M, Rodriguez-Prieto I (2006). Relationships of anti-predator escape and post-escape responses with body mass and morphology: a comparative avian study. Evolut Ecol Res.

[CR33] Franson JC, Pain DJ (2011) Lead in birds. USGS Staff-Published Research, vol 974. CRC Press, Boca Raton, FL, USA. https://digitalcommons.unl.edu/cgi/viewcontent.cgi?article=1984&context=usgsstaffpub

[CR34] Gangoso L, Alvarez-Lloret P, Rodríguez-Navarro AAB, Mateo R, Hiraldo F, Donazar JA (2009). Long-term effects of lead poisoning on bone mineralization in vultures exposed to ammunition sources. Environ Pollut.

[CR35] Geens A, Dauwe T, Bervoets L, Blust R, Eens M (2010). Haematological status of wintering great tits (*Parus major*) along a metal pollution gradient. Sci Total Environ.

[CR36] Goodchild CG, Beck ML, VanDiest I, Czesak FN, Lane SJ, Sewall KB (2021). Male zebra finches exposed to lead (Pb) during development have reduced volume of song nuclei, altered sexual traits, and received less attention from females as adults. Ecotoxicol Environ Saf.

[CR37] Gorissen L, Snoeijs T, Van Duyse E, Eens M (2005). Heavy metal pollution affects dawn singing behaviour in a small passerine bird. Oecologia.

[CR380] Grunst AS, Grunst ML, Thys B, Raap T, Daem N, Pinxten R, Eens M (2018). Variation in personality traits across a metal pollution gradient in a free-living songbird. Sci Total Environ.

[CR38] Grunst AS, Grunst ML, Thys B, Raap T, Daem N, Pinxten R, Eens M (2018). Variation in personality traits across a metal pollution gradient in a free-living songbird. Sci Total Environ.

[CR39] Harris T (2022) The physiological effects of high blood lead levels in the house sparrow. MSc thesis. Macquarie University, Sydney, NSW, AUS. https://figshare.mq.edu.au/articles/thesis/The_physiological_effects_of_high_blood_lead_levels_in_the_House_sparrow/21539379/1/files/38177226.pdf

[CR40] Hellou J (2011). Behavioural ecotoxicology, an “early warning” signal to assess environmental quality. Environ Sci Pollut Res.

[CR41] Herring G, Eagles-Smith CA, Bedrosian B, Craighead D, Domenech R, Langner HW, Parish CN, Shreading A, Welch A, Wolstenholme R (2018). Critically assessing the utility of portable lead analyzers for wildlife conservation. Wildl Soc Bull.

[CR42] Hossain S, Bhowmick S, Jahan S, Rozario L, Sarkar M, Islam S, Basunia MA, Rahman A, Choudhury BK, Shahjalal H (2016). Maternal lead exposure decreases the levels of brain development and cognition-related proteins with concomitant upsurges of oxidative stress, inflammatory response and apoptosis in the offspring rats. Neurotoxicology.

[CR43] Huang P, Sieving KE, St. Mary CM (2012). Heterospecific information about predation risk influences exploratory behavior. Behav Ecol.

[CR44] Ishii C, Nakayama SMM, Kataba A, Ikenaka Y, Saito K, Watanabe Y, Makino Y, Matsukawa T, Kubota A, Yokoyama K, Mizukawa H, Hirata T, Ishizuka M (2018). Characterization and imaging of lead distribution in bones of lead-exposed birds by ICP-MS and LA-ICP-MS. Chemosphere.

[CR45] Jackson BE, Evangelista DJ, Ray DD, Hedrick TL (2016). 3D for the people: multi-camera motion capture in the field with consumer-grade cameras and open source software. Biol Open.

[CR47] Klerks PL, Weis JL (1987). Genetic adaptation to heavy metals in aquatic organisms: a review. Environ Pollut.

[CR48] Kluen E, Siitari H, Brommer JE (2014). Testing for between individual correlations of personality and physiological traits in a wild bird. Behav Ecol Sociobiol.

[CR49] Lee JW, Choi H, Hwang UK, Kang JC, Kang YJ, Kim KI, Kim JH (2019). Toxic effects of lead exposure on bioaccumulation, oxidative stress, neurotoxicity, and immune responses in fish: a review. Environ Toxicol Pharmacol.

[CR50] Levin R, Brown MJ, Kashtock ME, Jacobs DE, Whelan EA, Rodman J, Schock MR, Padilla A, Sinks T (2008). Lead exposures in US children, 2008: implications for prevention. Environ Health Perspect.

[CR51] Lima SL (1993). Ecological and evolutionary perspectives on escape from predatory attack: a survey of North American birds. Wilson Bull.

[CR52] Marx SK, Rashid S, Stromsoe N (2016). Global-scale patterns in anthropogenic Pb contamination reconstructed from natural archives. Environ Pollut.

[CR53] McClelland SC, Durães-Ribeiro R, Mielke HW, Finkelstein ME, Gonzales CR, Komdeur J, Derryberry E, Saltzberg EB, Karubian J (2019). Sub-lethal exposure to lead is associated with heightened aggression in an urban songbird. Sci Total Environ.

[CR54] McCowan LSC, Mainwaring MC, Prior NH, Griffith SC (2015). Personality in the wild zebra finch: exploration, sociality, and reproduction. Behav Ecol.

[CR55] Miller TE, Golemboski KA, Ha RS, Bunn T, Sanders FS, Dietert RR (1998). Developmental exposure to lead causes persistent immunotoxicity in Fischer 344 rats. Toxicol Sci.

[CR56] Mogren CL, Trumble JT (2010). The impacts of metals and metalloids on insect behavior. Entomol Exp Appl.

[CR57] Monchanin C, Blanc-Brude A, Drujont E, Negahi MM, Pasquaretta C, Silvestre J, Baqué D, Elger A, Barron AB, Devaud JM, Lihorreau M (2021). Chronic exposure to trace lead impairs honey bee learning. Ecotoxicol Environ Saf.

[CR58] Mouneyrac C, Mastain O, Amiard JC, Amiard-Triquet C, Beaunier P, Jeantet AY, Smith BD, Rainbow PS (2003). Trace-metal detoxification and tolerance of the estuarine worm *Hediste diversicolor* chronically exposed in their environment. Mar Biol.

[CR59] Mouneyrac C, Leung PT, Leung KM, Amiard-Triquet C, Rainbow P, Roméo M (2011). Cost of tolerance. Tolerance to environmental contaminants-2011.

[CR60] Nam DH, Rutkiewicz J, Basu N (2012). Multiple metals exposure and neurotoxic risk in bald eagles (*Haliaeetus leucocephalus*) from two Great Lakes states. Environ Toxicol Chem.

[CR61] Neal AP, Guilarte TR (2010). Molecular neurobiology of lead (Pb^2+^): effects on synaptic function. Mol Neurobiol.

[CR62] Nixdorf WL, Taylor DH, Isaacson LG (1997). Use of bullfrog tadpoles (*Rana catesbeiana*) to examine the mechanisms of lead neurotoxicity. Am Zool.

[CR63] Owen JC (2011). Collecting, processing, and storing avian blood: a review. J Field Ornithol.

[CR64] Pain DJ, Mateo R, Green RE (2019). Effects of lead from ammunition on birds and other wildlife: a review and update. Ambio.

[CR65] Peterson EK, Buchwalter DB, Kerby JL, LeFauve MK, Varian-Ramos CW, Swaddle JP (2017). Integrative behavioral ecotoxicology: bringing together fields to establish new insight to behavioral ecology, toxicology, and conservation. Curr Zool.

[CR650] Pobleté EK, Botero-Delgadillo DB, Vásquez JL (2021). Risk-taking behaviour relates to timing of breeding in a sub-Antarctic rainforest bird. Ibis.

[CR66] Provini P, Tobalske BW, Crandell KE, Abourachid A (2012). Transition from leg to wing forces during take-off in birds. J Exp Biol.

[CR67] R Core Team (2021) R: a language and environment for statistical computing. Published online 2020. https://www.R-project.org/

[CR68] Roux KE, Marra PP (2007). The presence and impact of environmental lead in passerine birds along an urban to rural land use gradient. Arch Environ Contam Toxicol.

[CR69] Sato H, Ishii C, Nakayama SMM, Ichise T, Saito K, Watanabe Y, Ogasawara K, Torimoto R, Kobayashi A, Kimura T, Nakamura Y, Yamagishi J, Ikenaka Y, Ishizuka M (2022). Behavior and toxic effects of Pb in a waterfowl model with oral exposure to Pb shots: investigating Pb exposure in wild birds. Environ Pollut.

[CR70] Scott GR, Sloman KA (2004). The effects of environmental pollutants on complex fish behaviour: integrating behavioural and physiological indicators of toxicity. Aquat Toxicol.

[CR71] Seewagen CL (2020). The threat of global mercury pollution to bird migration: potential mechanisms and current evidence. Ecotoxicology.

[CR72] Swaddle JP, Witter MS (1997). The effects of molt on the flight performance, body mass, and behavior of European starlings (*Sturnus vulgaris*): an experimental approach. Can J Zool.

[CR73] Swaddle JP, Williams EV, Rayner JMV (1999). The effect of simulated flight feather moult on escape take-off performance in starlings. J Avian Biol.

[CR74] Swaileh KM, Sansur R (2006). Monitoring urban heavy metal pollution using the house sparrow (*Passer domesticus*). J Environ Monit.

[CR75] Thady RG, Emerson LC, Swaddle JP (2022). Evaluating acoustic signals to reduce avian collision risk. PeerJ.

[CR76] Vallverdú-Coll N, Mougeot F, Ortiz-Santaliestra ME, Castaño C, Santiago-Moreno J, Mateo R (2016). Effects of lead exposure on sperm quality and reproductive success in an avian model. Environ Sci Technol.

[CR77] Vallverdú-Coll N, Mougeot F, Ortiz-Santaliestra ME (2019). Immunotoxic effects of lead on birds. Sci Total Environ.

[CR78] Veasey JS, Houston DC, Metcalfe NB (2001). A hidden cost of reproduction: the trade-off between clutch size and escape take-off speed in female zebra finches. J Anim Ecol.

[CR79] Weber DN (1993). Exposure to sublethal levels of waterborne lead alters reproductive behavior patterns in fathead minnows (*Pimephales promelas*). Neurotoxicology.

[CR80] White JH, Heppner JJ, Ouyang JQ (2022). Increased lead and glucocorticoid concentrations reduce reproductive success in house sparrows along an urban gradient. Ecol Appl.

[CR81] Williams RJ, Holladay SD, Williams SM, Gogal RM (2018) Environmental lead and wild birds: a review. In DeVoogt P (ed) Reviews of environmental contamination and toxicology, vol 245, pp 157–180. Springer International Publishing. Cham, Switzerland. http://ndl.ethernet.edu.et/bitstream/123456789/35094/1/pdf%2C74#page=168 10.1007/398_2017_9 29038944

[CR82] Witter MS, Cuthill IC, Bonser RHC (1994). Experimental investigations of mass-dependent predation risk in the European starling, *Sturnus vulgaris*. Anim Behav.

[CR83] Xie L, Klerks PL (2004). Fitness cost of resistance to cadmium in the least killifish (*Heterandria formosa*). Environ Toxicol Chem Int J.

[CR84] Zahor DL (2021) Bioaccumulation of lead (Pb) in songbirds following the Flint, Michigan, Drinking Water Crisis. MS thesis. Eastern Michigan University, Ypsilanti, MI, USA. https://www.proquest.com/docview/2572918334?pq-origsite=gscholar&fromopenview=true&sourcetype=Dissertations%20&%20Theses

[CR85] Zheng Y, Zhang O, Jing L, Fei Y, Zhao H (2022) The effects of chronic lead exposure on testicular development of Japanese quail (*Coturnix japonica*): histopathological damages, oxidative stress, steroidogenesis disturbance, and hypothalamus-pituitary-testis axis disruption. In: Biological trace element research, pp 1–15. 10.1007/s12011-022-03436-8 10.1007/s12011-022-03436-8 36210404

[CR86] Zou RX, Gu X, Ding JJ, Wang T, Bi N, Niu K, Ge M, Chen XT, Wang HL (2020). Pb exposure induces an imbalance of excitatory and inhibitory synaptic transmission in cultured rat hippocampal neurons. Toxicol in Vitro.

